# Association between cerebrospinal fluid pressure and cognition in patients with Alzheimer’s disease and Lewy body dementia

**DOI:** 10.1186/s12883-023-03502-1

**Published:** 2024-01-19

**Authors:** Xia Yang, Jinghuan Gan, Yong Ji

**Affiliations:** 1 Department of Neurology, Beijing Tiantan Hospital, China National Clinical Research Center for Neurological Diseases, Capital Medical University, Beijing, China; 2 Department of Neurology, Beijing Friendship Hospital, Capital Medical University, Beijing, China; 3 Department of Neurology, Tianjin Dementia Institute, Tianjin Key Laboratory of Cerebrovascular and Neurodegenerative Diseases, Tianjin Huanhu Hospital, Tianjin, China

**Keywords:** Cerebrospinal fluid pressure, Alzheimer’s disease, Lewy body disease, Cerebrospinal fluid (CSF)/serum albumin ratio

## Abstract

**Background:**

The relationship between cerebrospinal fluid pressure (CSFP) and cognition has received little research attention. The purpose of this study was to explore the relationship between CSFP and cognition in patients with Alzheimer’s disease (AD) and patients with Lewy body dementia (LBD).

**Method:**

We included 178 participants, including 137 patients with AD and 41 patients with LBD (including dementia with Lewy bodies (DLBs) and Parkinson’s disease dementia (PDD)). CSFP was measured by lumbar puncture, and a patient-reported history and laboratory test data were collected. Logistic and linear regression analyses were used to evaluate the associations between CSFP and cognition, the cerebrospinal fluid (CSF) / serum albumin ratio (Qalb), and CSF biomarkers of AD.

**Results:**

The mean age of the included patients was 63.58 ± 8.77 years old, and the mean CSFP was 121 ± 33.72 mmH2O. A total of 76.9% of the patients had a CSFP distribution of [90–170) mmH2O, 46 patients (25.8%) had severe dementia, 83 patients (46.6%) had moderate dementia, 28 patients (15.7%) had mild dementia, and 21 patients (11.8%) had mild cognitive impairment (MCI) (including 16 patients with MCI due to AD and 5 patients with MCI due to LBD). In all patients (p value < 0.001) and in patients with AD (p value = 0.01), the mean cerebrospinal fluid pressure (CSFP) was higher in patients with MCI than in patients with dementia. In multivariate analysis, in all patients (OR: 6.37, 95% confidential interval (CI): 1.76–23.04, p = 0.005) and patients with AD (odds ratio (OR): 5.43, 95% CI: 1.41–20.87, p = 0.005), a CSFP in the lowest quartile ([50–90) mmH2O) was associated with a higher level of severe dementia than a CSFP in the highest quartile ([170–210) mmH2O). In addition, there was a significant linear correlation between CSFP and the Mini-Mental State Examination (MMSE) score in all patients with dementia (r = 0.43, p = 0.04, Durbin-Watson test (D-W test) = 0.75).

**Conclusion:**

In patients with AD, the mean cerebrospinal fluid pressure was higher in patients with MCI than in patients with dementia, and the decrease in CSFP was related to a more serious dementia level. However, no such relationship was found in patients with LBD.

**Supplementary Information:**

The online version contains supplementary material available at 10.1186/s12883-023-03502-1.

## Introduction

Dementia has become a significant cause of disability in individuals over 65 years of age worldwide [1], and the number of patients with dementia in China accounts for approximately 25% of the entire population with dementia worldwide [2], which poses an enormous challenge to public health. In a 1994 Medical Hypotheses paper, it was proposed that high intracranial pressure (ICP) might play a role in the pathogenesis of Alzheimer’s disease (AD) [3]. Longitudinal studies have pointed out the cumulative effect of intermittent ICP elevation on choroid plexus and meningeal damage, reduced cerebrospinal fluid clearance of neurotoxins such as amyloid-β (Aβ) protein and direct damage to the hippocampus [4, 5]. The above observations further argue for the role of elevated ICP in the pathogenesis of AD. The paper further proposed that this pressure factor may be missing in the later stage of AD. Additionally, patients with idiopathic intracranial hypertension (a cerebrospinal fluid pressure (CSFP) > 200–250 mmH2O) suffer from cognitive deficits [6], and importantly, cognitive deficits can improve with time and reduced ICP [7]. However, among people with cognitive impairment and further disease progression, there are no relevant studies to explain the relationship between cognition and CSFP; moreover, the current study was limited to patients with AD. We investigated the relationship between CSFP and cognition in patients with Lewy body dementia (LBD) and patients with AD and the possible reasons for the relationship.

## Materials and methods

### Participant recruitment

The study included 178 hospitalized patients diagnosed with AD (n = 137) and LBD (n = 41) recruited from the Department of Cognitive Disorders of Beijing Tiantan Hospital, Capital Medical University from December 2019 to April 2023. Dementia and mild cognitive impairment (MCI) were diagnosed according to the criteria outlined in the Diagnostic and Statistical Manual of Mental Disorders-Five Edition [8]. Probable AD was diagnosed according to the criteria of the National Institute on Aging-Alzheimer’s Association (NIA-AA) workgroup or a cerebrospinal fluid (CSF) test for neuropathological biomarkers of AD (*n* = 105) [9]. LBD patients included patients with dementia with Lewy bodies (DLBs) and Parkinson’s disease dementia (PDD). The patients with probable DLB met the consensus criteria for probable DLB (2017 version) [10], and the patients with probable PDD met the clinical criteria for probable PDD developed by the Movement Disorder Society in 2007 [11]. Probable Parkinson’s disease with mild cognitive impairment (PD-MCI) was diagnosed by the diagnostic criteria developed by the Movement Disorder Society Task Force for a level I or level II diagnosis [12]. Since a consensus regarding the criteria for probable mild cognitive impairment with Lewy bodies (MCI-LB) was in progress at the time of first diagnosis, probable MCI-LB was initially defined with a combination of MCI criteria using Petersen’s criteria developed in 2011 [13] and DLB criteria developed by McKeith in 2017 [10] with an MMSE score ≥ 20 and a CDR score ≥ 0.5 [14]. International consensus suggests that DLB should be diagnosed when cognitive impairment precedes parkinsonism or begins within a year of parkinsonism diagnosis, and PDD should be diagnosed when a parkinsonism diagnosis precedes cognitive impairment by more than 1 year.

To ensure that patients did not suffer from comorbidities that could affect CSFP, a series of exclusion criteria were used, including inflammation and tumors of the brain as well as the spinal cord, cerebral hemorrhage, spinal canal obstruction, radiculopathy, and extreme weakness due to heart failure, liver failure, renal failure, or cancer. We also excluded patients with idiopathic normal pressure hydrocephalus [15], idiopathic intracranial hypertension [6] or idiopathic hypocranial pressure [16]. To avoid the effect of drugs that affect cerebrospinal fluid circulation, such as vinpocetine, mannitol, diuretic drugs, and glipizide, such drugs were discontinued for at least 24 h prior to lumbar puncture.

### Clinical assessment

We collected patient demographic and clinical information, including age, sex, education level, smoking status (with a history of smoking ≥ 5 cigarettes per day for > 2 years), alcohol consumption (with a history of drinking an alcoholic beverage ≥ 1 time per week for > 2 years) [17] and history of hypertension and diabetes, heart disease, stroke, and hyperlipidemia by reviewing the patients’ records. Hyperlipidemia was defined as levels of serum cholesterol ≥ 5.20 mmol/L, triglycerides ≥ 1.7 mmol/L, high-density lipoprotein cholesterol ≤ 1.04 mmol/L, or low-density lipoprotein cholesterol ≥ 3.61 mmol/L or previously diagnosed hyperlipidemia [18]. Hypertension was defined as an average systolic blood pressure of at least 140  mmHg, an average diastolic blood pressure of at least 90  mmHg, or self-reported use of an antihypertensive drug 2 weeks before the visit [19]. Type 2 diabetes mellitus was defined as a self-reported previous diagnosis, the use of diabetic medications, or a hemoglobin A1c level of 6.5% or greater [20]. A history of heart disease, including cardiovascular diseases, heart failure, arrhythmia, valvular heart disease, and congenital heart disease, was defined as a self-reported previous diagnosis and/or the use of related medications based on clinical records. Stroke history, including ischemic stroke and intracerebral hemorrhage stroke, was defined as clinical presentation with confirmation by computed tomography (CT) or MRI based on clinical records [19]. Considering that patients with dementia may not be able to provide reliable information due to memory impairment, confirmation was obtained from a caregiver who were aware of the patient’s disease status by asking for basic clinical information about the patient.

We also collected data on cerebrospinal fluid (CSF)/serum albumin ratio (Qalb) values for 148 patients and Aβ1–42, Aβ1–40, p-tau181, and t-tau values for 132 patients (including 27 patients with LBD and 105 patients with AD).

Sleep disorders mainly included insomnia, excessive daytime sleepiness (EDS), rapid eye movement sleep behavior disorder (RBD), and obstructive sleep apnea hypopnea syndrome (OSAHA). The Pittsburgh Sleep Quality Index (PSQI) was used to evaluate sleep quality during the last month, and we considered a patient to have poor sleep quality in the last month when the PSQI total score was > 5 points [21]. RBD and hypersomnolence were assessed by the Rapid Eye Movement Sleep Behavior Disorder Screening Questionnaire and Epworth Sleepiness Scale (ESS); when the RBD screening questionnaire score was > 5 points and the ESS total score was ≥ 10 points [22], RBD and EDS were considered to exist [23]. OSAHA was assessed by the Berlin Questionnaire, and the presence of OSA was indicated when there were greater than or equal to 2 parts with a score greater than or equal to 2 points [24, 25]. Anxiety and depression status was assessed by using the Hamilton Depression Inventory (HAMD) and Hamilton Anxiety Inventory (HAMA) scales. When the HAMA score was > 17/56 or the HAMD score was ≥ 8/52, we considered the presence of possible anxiety or depression [26, 27].

### Neuropsychological assessments

Neuropsychological assessments were performed on the same day as the lumbar puncture. The Mini-Mental State Examination-Chinese version (C-MMSE) [28], the Montreal Cognitive Assessment (MoCA) and the Clinical Dementia Rating (CDR) [29] were performed, and the C-MMSE and the CDR scale were used to evaluate global cognitive function and the severity of cognitive impairment in all patients. The MoCA, with a cutoff point of 25/26, is the recommended cognitive screening tool for mild cognition and mild cognitive impairment (MCI) [30]. The CDR is a 5-point scale; scores of 0.0 (no dementia), 0.5 (MCI), 1.0 (mild), 2.0 (moderate), and 3.0 (severe) are possible [28].

### Laboratory measurements

#### Collection of cerebrospinal fluid and blood

Between 7 and 10 a.m., the patient was placed in the left lateral recumbent position (spine in a straight line on the horizontal plane, head in a neutral position, knees bent, and the midline of the spine at the same height as the patient’s head) [31]. The L3 to L4 or L4 to L5 intervertebral space was selected. A 20-gauge (0.9 mm) lumbar puncture needle was used for lumbar puncture, and a disposable plastic manometry tube was used to measure cerebrospinal fluid pressure. The patient was asked to extend the leg slightly at the hip while avoiding coughing and other Valsalva movements. When the CSF level did not rise any further, the patient was placed in a calm state while breathing quietly, and the height of the lowest part of the top bend of the fluid column was read [32]. It took approximately 1 min to record the CSFP. Then, 10 mL of mid-cerebrospinal fluid was collected in a sterile blank tube. After lumbar puncture, a blood sample was drawn through venipuncture into a 6-mL plastic vacuum tube containing EDTA, and the blood was immediately centrifuged and stored in a polypropylene tube at -80 °C until use. Routine and biochemical test results were obtained in 2–3 h. CSF biomarkers of AD were detected in approximately 2–3 days. All analyses of blood and CSF samples were performed using commercial and validated instruments and kits at the Clinical Neurochemistry Laboratory at Beijing Tiantan Hospital, Beijing, China. In addition, before lumbar puncture, blood pressure was measured twice on the right upper arm with an electronic sphygmomanometer (Omron HEM-730; Omron Corporation, Kyoto, Japan) with a 1-minute interval between measurements, and the mean systolic and diastolic blood pressure values were calculated and recorded [19, 20].

#### Measurement of cerebrospinal fluid and blood

Immunoturbidimetric assays were performed to detect cerebrospinal fluid albumin levels, and serum albumin levels were analyzed using the absorption method. The permeability of the blood‒brain barrier (BBB) was characterized by the CSF albumin to serum albumin ratio [33]. CSF Aβ1–42 (RE59661, IBL International, Hamburg, Germany), Aβ1–40 (RE59651, IBL International, Hamburg, Germany), t-tau (RE59631, IBL International, Hamburg, Germany), and p-tau181 (30,121,609, IBL International, Hamburg, Germany) concentrations were quantified using commercial enzyme-linked immunosorbent assays (ELISAs) according to the manufacturer’s protocol. CSF cutoff values for Aβ positivity or Aβ negativity were an Aβ1–42 value < 550 pg/mL and/or an Aβ1–42/Aβ1–40 ratio value ≤ 0.05. CSF cutoff values for tau positivity were a p-tau181 value > 50 pg/mL and/or a t-tau value > 399 pg/mL, and all cutoff values were set based on the accumulation of previous experimental data from Kindstar Global Genetic Technology Co., LTD [19, 20].

### Statistical analysis

Descriptive analyses were conducted using frequencies for qualitative variables and the mean [± standard deviation (SD)] or median (Q_25_,_75_) for quantitative variables. For qualitative variables, between-group analysis was performed using the chi-square test or Fisher’s exact test (when cell expectation frequencies were less than 5), and for continuous variables, intergroup analysis was performed using one-way variance (normal distribution) or Kruskal‒Wallis H test (nonnormal distribution). Logistic regression was used to analyze the correlation between CSFP and dementia severity. Linear regression [along with the 95% confidence interval (CI)] and the Durbin-Watson (D-W) test were used to describe the correlation between CSFP and MMSE score. Goodness-of-fit tests (Pearson and deviance tests) were used to estimate discrete parameters and to test the adequacy of the model. The Durbin-Watson (D-W) test was used to analyze the correlation between CSFP and cerebrospinal fluid biomarkers of AD, and Qalb values, sex, age and vascular risk factors were adjusted for. The comparisons among the amyloid/tau/neurodegeneration (ATN) framework were conducted using the Kruskal‒Wallis H Test and Bonferroni corrections were also applied. When the p value was < 0.0083, we considered that there was a difference between the two groups. All analyses were performed using SPSS version 27.0 (SPSS, Inc., Chicago, IL, USA). All reported *p* values were two-sided, and the results were considered statistically significant when the *p* value was < 0.05.

## Results

### Sample characteristics

Demographic and clinical characteristics are shown in Table 1. The mean age of the included patients was 63.58 ± 8.77 years, and the mean CSFP was 121 ± 33.72 mmH2O. Except for age, sex, and history of heart disease, significant differences were not found in the course of disease, educational level, MMSE score, CSFP, Qalb value or other characteristics between the two groups.

**Table 1:** Demographic and clinical characteristics and laboratory test results of patients with different cognitive disorders

	All patients (n = 178)	AD patients (n = 137)	DLB LBD patients (n = 41)	P value
Age (years)	63.58 ± 8.77	62.23 ± 8.68	68.07 ± 7.55	**<0.001**
Female sex, n (%)	103 (57.9%)	87 (63.5%)	16 (39.0%)	**0.007**
Course of disease (years)	3.33 ± 2.87	3.43 ± 3.04	2.99 ± 2.19	0.39
Education level (years) SBP (mmHg)	9.60 ± 4.26 121 ± 16.13	9.65 ± 4.25 121 ± 15.72	9.44 ± 4.34 122 ± 17.65	0.77 0.84
Regular smoking, n (%)	48 (27.0%)	34 (24.8%)	14 (34.1%)	0.31
Regular alcohol consumption, n (%)	39 (21.9%)	28 (20.4%)	11 (26.8%)	0.39
Heart disease, n (%)	15 (8.4%)	15 (10.9%)	0	**0.02**
Stroke, n (%)	16 (9.0%)	11 (8.0%)	5 (12.2%)	0.53
Hypertension, n (%)	60 (33.7%)	45 (32.8%)	15 (36.6%)	0.71
Diabetes, n (%)	30 (16.9%)	25 (18.2%)	5 (12.2%)	0.47
Hyperlipidemia, n (%)	72 (40.4%)	57 (41.6%)	15 (36.6%)	0.59
Anxiety state, n (%)	52 (29.2%)	38 (27.7%)	13 (31.7%)	0.43
Depression state, n (%)	57 (32.0%)	42 (30.7%)	15 (36.6%)	0.56
Sleep disorder, n (%)	87 (48.9%)	64 (46.7%)	23 (56.1%)	0.37
CSFP level(mmH2O)	121 ± 33.72	122 ± 32.56	121 ± 37.76	0.88
MMSE Score MoCA Score CDR	14.77 ± 7.13 10.33 ± 6.21 1.92 ± 0.82	14.70 ± 7.20 10.48 ± 6.15 1.94 ± 0.82	15.00 ± 6.99 9.80 ± 6.46 1.86 ± 0.84	0.81 0.54 0.61
Qalb	6.82 ± 5.23	6.76 ± 5.67	6.85 ± 3.63	0.80

AD, Alzheimer’s disease; LBD, Lewy body dementia; MMSE, Mini-Mental State Examination; CSFP, cerebrospinal fluid pressure; MoCA, Montreal Cognitive Assessment; Qalb, cerebrospinal fluid (CSF)/serum albumin ratio; SBP, systolic blood pressure (before lumbar puncture)

Bold: Significant differences were found in age (*p* < 0.001), sex (*p* = 0.007), and history of heart disease (*p* = 0.02) between patients with AD and LBD

### Cerebrospinal fluid pressure and cognitive level

#### Subject characteristics

Among the patients, 46 (25.8%) had severe dementia, 83 (46.6%) had moderate dementia, 28 (15.7%) had mild dementia, and 21 (11.8%) had MCI (including 16 patients with MCI due to AD and 5 patients with MCI due to LBD). As shown in Appendix 1, there was a significant correlation between CSFP quartiles and MMSE scores in all participants and patients with AD. History of diabetes differed by CSFP quartile in a quarter of all participants and patients of AD, and history of stroke and Qalb values differed by CSFP quartile in patients with AD. We also found that Aβ1–42 and Aβ1–40 values differed by CSFP quartile in patients with LBD.

AD, Alzheimer’s disease; LBD, Lewy body dementia; MMSE, Mini-Mental State Examination; CSFP, cerebrospinal fluid pressure; Qalb, cerebrospinal fluid (CSF)/serum albumin ratio; SBP: systolic pressure (before lumbar puncture).

a: there was an intergroup difference between patients with CSFP values in the lowest quartile and the second quartile, b: there was an intergroup difference between patients with CSFP values in the lowest quartile and the third quartile, c: there was an intergroup difference between patients with CSFP values in the lowest quartile and the fourth quartile, d: there was an intergroup difference between patients with CSFP values in the second quartile and the third quartile, e: there was an intergroup difference between patients with CSFP values in the second quartile and the fourth quartile, f: there was an intergroup difference between patients with CSFP values in the third quartile and the fourth quartile.

#### Associations between CSFP and cognitive performance

The correlation between CSFP and the severity of dementia was evaluated. The results are shown in Table 2. In the multivariate analysis controlling for sex, age, education level, course of disease and vascular risk factors, in all patients (odds ratio (OR): 6.37, 95% CI: 1.76–23.04, p = 0.005) and patients with AD (OR: 5.43, 95% CI: 1.41–20.87, p = 0.005), a CSFP level in the lowest quartile was associated with more severe dementia, and this correlation was not found in patients with DLB. Pearson and chi-square deviation tests did not show excessive dispersion (p > 0.05) in the logistic regression model.

**Table 2:** Odds ratios and 95% confidence intervals for dementia based on CSFP

	Model 1		Model 2		Model 3		Model 4	
	OR	95% CI	OR	95% CI	OR	95% CI	OR	95% CI
**All patients**								
[50–90)	5.76^**^	1.77–18.75	7.92^**^	2.23–27.19	7.21^**^	2.01–25.75	6.37^**^	1.76–23.04
[90–130)	6.63^**^	2.39–18.35	6.99^**^	2.46–19.88	7.51^**^	2.59–21.78	6.77^**^	2.31–19.81
[130–170]	3.11^*^	1.12–8.64	3.15^*^	1.11–8.96	3.15^*^	1.09–9.08	3.40^*^	1.17–9.92
[170–210)	1		1		1		1	
**AD patients**								
[50–90)	8.55^**^	1.94–37.60	9.08^**^	2.10-39.19	10.27^**^	2.48–42.53	5.43^**^	1.41–20.87
[90–130)	6.38^**^	1.82–22.33	6.76^**^	1.96–23.26	6.22^**^	1.88–20.51	5.00^**^	1.56–15.98
[130–170)	3.72^*^	1.06–13.02	3.26	0.95–11.17	3.23	0.97–10.79	2.51	0.77–8.13
[170–210)	1		1		1		1	
**LBD patients**								
[50–90)	7.51	0.60-93.59	2.38	0.13–41.15	4.48	0.15-128.05	2.91	0.06-127.01
[90–130)	18.57^*^	2.05-167.58	9.64	0.93–99.32	18.51^*^	1.18-290.13	25.63	0.97-676.92
[130–170)	6.82	0.80-57.93	2.06	0.19–22.35	3.33	0.21–53.05	5.51	0.20-149.95
[170–210)	1		1		1		1	

*Note*: Model 1: Unadjusted. Model 2: Adjusted for age, sex, course of disease, and education level. Model 3: Adjusted for age, sex, course of disease, education level, smoking status, alcohol consumption, history of stroke, hyperlipidemia, heart disease, diabetes, and hypertension. Model 4: Adjusted for age, sex, course of disease, education level, smoking status, alcohol consumption, history of stroke, hyperlipidemia, heart disease, diabetes, hypertension, anxiety, depression, and sleep disorders

AD, Alzheimer’s disease; LBD, Lewy body dementia

**p < 0.01 and *p < 0.05 compared with CSFP values in the highest quartile ([170–210) mmH2O)

As shown in Table 3, in all patients, the mean cerebrospinal fluid pressure was higher in patients with MCI (145 ± 27.77) than in patients with dementia (118 ± 33.21), with a p value < 0.001, and in patients with AD (p value = 0.01), in patients with LBD (p value = 0.004), the mean cerebrospinal fluid pressure was also higher in patients with MCI than in patients with dementia.

**Table 3:** Cerebrospinal fluid pressure in patients with different cognitive levels

	Population		P value
**All patients**			
	MCI	dementia	
N	21	157	
CSFP	145 ± 27.77	118 ± 33.21	<0.001
**AD**			
	MCI	dementia	
N	16	121	
CSFP	140 ± 26.01	119 ± 32.70	0.01
**LBD**			
	MCI	dementia	
N	5	36	
CSFP	165 ± 26.92	115 ± 35.15	0.004

AD, Alzheimer’s disease; LBD, Lewy body dementia; CSFP, cerebrospinal fluid pressure

**Fig. 1:**
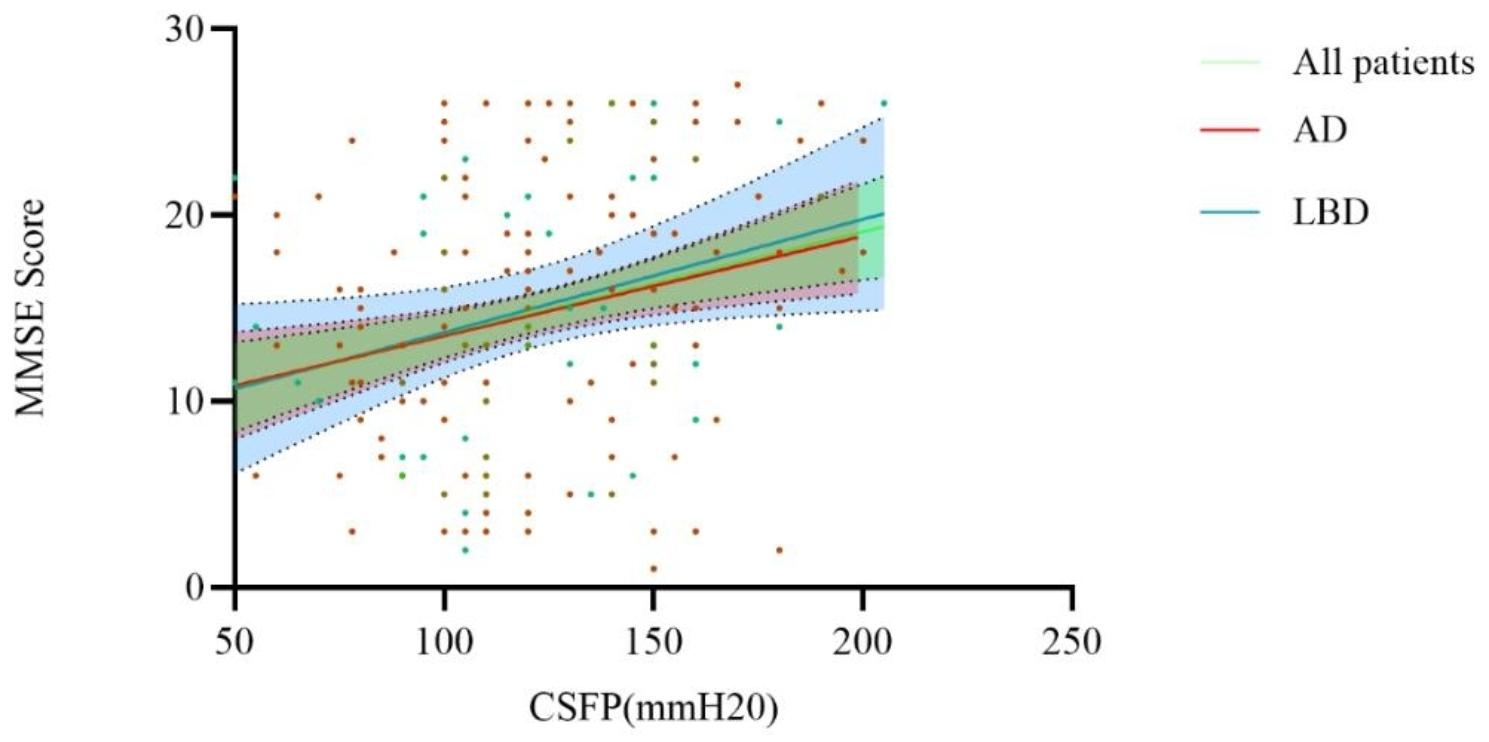
Linear regression and 95% CI between CSFP and cognition in patients with different cognitive disorders. AD, Alzheimer’s disease; 95% CI, 95% confidence interval; LBD, Lewy body dementia; CSFP, cerebrospinal fluid pressure; MMSE, Mini-Mental State Examination

As shown in Fig. 1, in the population with dementia, after controlling for sex, age, education level, course of disease and vascular risk factors, there was a significant positive correlation between CSFP and MMSE scores in all patients (r = 0.43, p = 0.04, D-W test = 0.75). However, this linear correlation disappeared after adjustment in patients with LBD ((r = 0.46, p = 0.62, D-W test = 2.01) and in AD patients (r = 0.45, p = 0.06, D-W test = 0.87).

### Possible reasons for the influence of cerebrospinal fluid on cognition

The results are shown in Table 4. The results showed that CSFP was negatively associated with the Qalb values in all patients (n = 148) (B = -0.03, 95% CI: -0.05 - -0.01, p = 0.006) and in the patients with AD (n = 113) (B = -0.04, 95% CI: -0.07 - -0.01, p = 0.006) but not the levels of Aβ1–42, Aβ1–40, p-tau181, and t-tau. In the patients with LBD, we found that there was a linear relationship between CSFP and the levels of Aβ1–42 (B = 4.22, 95% CI: 0.06–8.42, *p* = 0.04).

**Table 4:** Associations between CSFP and CSF biomarkers of AD and Qalb values

	Linear regressions					
	B	SE	Beta	t	P value	B 95% CI
All patients						
Aβ1–42 (pg/ml)	1.21	0.99	0.10	1.21	0.23	-0.77-3.17
Aβ1–40 (pg/ml)	10.55	13.77	0.06	0.76	0.44	-16.71-37.81
t-tau (pg/ml)	13.58	9.03	0.13	1.50	0.13	-4.29-31.29
p-tau181 (pg/ml)	-0.11	0.31	0.03	0.33	0.73	-0.51-0.72
Aβ1–42/Aβ1–40	-3.62	8.7 × 10-5	-0.03	-0.41	0.68	-2.1 × 10-4-1.3 × 10-4
p-tau181/Aβ1–42	2.8 × 10-4	0.001	0.02	0.26	0.79	-0.002-0.002
Qalb	-0.03	0.01	-0.21	-2.79	**0.006**	-0.05-0.01
**AD patients**						
Aβ1–42 (pg/ml)	0.94	1.15	0.07	0.81	0.41	-1.35-3.23
Aβ1–40 (pg/ml)	16.93	16.53	0.10	1.02	0.31	-15.88-49.74
t-tau (pg/ml)	16.08	11.56	0.13	1.39	0.16	-6.87-39.03
p-tau181 (pg/ml)	0.06	0.40	0.01	0.16	0.86	-0.72-0.86
Aβ1–42/Aβ1–40	1.2 × 10-4	1.0 × 10-4	-0.12	-1.26	0.21	-3.2 × 10-4-7.2 × 10-5
p-tau181/Aβ1–42	2.8 × 10-4	0.001	0.02	0.21	0.83	-0.002-0.003
Qalb	-0.04	0.15	-0.24	-2.78	**0.006**	-0.07-0.01
**LBD patients**						
Aβ1–42 (pg/ml)	4.22	2.01	0.46	2.09	**0.04**	0.02–8.42
Aβ1–40 (pg/ml)	4.55	23.65	0.04	0.19	0.84	-44.78-53.89
t-tau (pg/ml)	1.69	1.71	0.21	0.99	0.33	-1.87-5.27
p-tau181 (pg/ml)	0.12	0.18	0.15	0.69	0.49	-0.25-0.51
Aβ1–42/Aβ1–40	3.0 × 10-4	1.8 × 10-4	0.36	1.59	0.12	-9.3 × 10-5-0.001
p-tau181/Aβ1–42	2.2 × 10-4	0.001	-0.06	-0.27	0.78	-0.002-0.001
Qalb	-0.01	0.01	-0.17	-0.86	0.39	-0.05-0.02

The linear regression models were analyzed after adjusting for sex, age, education level, course of disease, and MMSE score for CSF biomarkers of AD. For the Qalb, we adjusted for sex, age, education level, course of disease and vascular risk factors

AD, Alzheimer’s disease; LBD, Lewy body dementia; Aβ, β-amyloid; p-tau181, phosphorylated tau181; t-tau, total tau; Qalb, cerebrospinal fluid/serum albumin value

Bold: CSFP was negatively associated with Qalb values in all patients (*p* = 0.006) and the patients with AD (*p* = 0.006). And there was a linear ralationship between CSFP and the levels of Aβ1-42 in the patients with LBD (*p* = 0.04)

As shown in Table 5, the main effect of CSFP and amyloid/tau/neurodegeneration (ATN) status in all patients (n = 132) was statistically significant, but no differences were found between AD and LBD patients. Intragroup differences were not observed between patients with AD and LBD.

**Table 5:** Comparisons of CSFP and CSF AD neuropathological biomarkers according to the ATN framework

	A-T-	A + T-	A-T+	A + T+	P value
**All patients**					
N	4	16	28	84	
CSFP (mmH2O)	161 ± 46.97	129 ± 40.65	130 ± 33.04	114 ± 31.45	0.02
**AD**					
N		12	22	71	
CSFP (mmH2O)		137 ± 10.93	129 ± 7.03	117 ± 3.73	0.06
**LBD**					
N	4	4	6	13	
CSFP (mmH2O)	161 ± 46.97	106 ± 45.34	133 ± 36.19	103 ± 29.75	0.09

In all patients, after Bonferroni corrections, no differences were found between the two groups

ATN, amyloid tau neurodegeneration framework; AD, Alzheimer’s disease; LBD, Lewy body dementia

## Discussion

This is the first cross-sectional study of cerebrospinal fluid pressure and cognitive changes in patients with dementia and Alzheimer’s disease. In our study, after controlling for sex, age, education level, and vascular risk factors, reduced CSFP in AD patients was associated with more severe cognitive impairment, which is consistent with the hypothesis that more advanced AD is associated with reduced CSFP, as suggested by Peter Wostyn et al. [34]. Silverberg et al. reported that among AD patients, those with a mean CSFP of 249 ± 20 mmH2O were younger and scored higher on the Mattis Dementia Rating Scale (MDRS) than those with a mean CSFP of 103 ± 47 mmH2O [35–37], suggesting an association between lower cerebrospinal fluid pressure and poorer cognition. This hypothesis was further confirmed in our cross-sectional study. We also found that reduced CSFP was positively associated with higher MMSE scores.

Recent studies have found significantly lower levels of Aβ1–41 and significantly higher levels of tau in the vitreous humor of patients with glaucoma [38, 39]. Given the anatomical and functional similarities between the intraocular pressure (IOP) gap and the ICP gap, it can be hypothesized that increased pressure may lead to similar neurodegenerative mechanisms in both pressure gaps, which may be at least partially shared with AD. Moreover, repeated intermittent intracranial pressure elevation causing hippocampal neuronal damage and choroid plexus damage may be an early trigger of the neurogenic cascade response in AD. In our study, the mean cerebrospinal fluid pressure was higher in patients with mild cognitive impairment than in patients with dementia, both in all patients and in AD patients. In the early stages of cognitive impairment, cerebrospinal fluid pressure values were significantly higher in patients with AD than in patients with dementia, and we hypothesize that cerebrospinal fluid pressure is elevated in the early stages of the disease and may be a potential mechanism for disease onset.

It is well known that ICP depends on cerebrospinal fluid dynamics and cerebral blood circulation pressure. Changes in cerebrospinal fluid circulation have an impact on cerebrospinal fluid pressure [40]. The blood‒brain barrier (BBB) is located between the brain parenchyma and the vascular system. It is a highly selective semipermeable structural and chemical barrier that ensures the stability of the internal brain environment and prevents the invasion of brain tissue by foreign bodies, and it is also crucial for cerebrospinal fluid circulation [41]. There is also a correlation between blood‒brain barrier dysfunction and cognitive impairment. Blood‒brain barrier dysfunction can lead to neuroinflammation and oxidative stress, which ultimately promote Aβ production and affect the failure of Aβ transfer to the peripheral circulation [42]. Alterations in Qalb values are considered a reliable standard surrogate marker of blood‒brain barrier integrity, which was found to be increased in patients with Parkinson’s disease and Alzheimer’s disease (AD) compared to healthy individuals [43, 44]. In our study, after correcting for confounders such as age, sex, and course of disease, we found a negative linear relationship between CSFP and Qalb values in all patients and in patients with AD but not in patients with LBD. However, we found that there was a linear relationship between CSFP and the levels of Aβ1–42. In addition, we found that in all patients, CSFP was different in amyloid protein/tau/neurodegeneration (ATN), with the lowest value in A + T + patients and the highest value in A-T- patients. Although this difference disappeared in patients with AD after adjusting for risk factors, cerebrospinal fluid pressure was still the lowest in A + T + patients. It has been shown that patients with AD exhibit lower CSFP than healthy controls, correlating with CSF Aβ1–42 levels [45]. In addition, it was found that the CSF production rate was significantly lower in AD patients than in PD patients [46], and a reduced CSF production rate also has an impact on CSFP, which reduces cerebrospinal fluid circulation and increases the deposition of pathological markers, which affects the cognitive level of patients. Therefore, we speculate that decreased cerebrospinal fluid (CSF) pressure affects blood‒brain barrier permeability, influences cerebrospinal fluid circulation, and increases the deposition of pathological markers of AD, which can affect patients’ cognitive levels.

In patients with LBD, we did not find a correlation between CSFP and cognition or the blood‒brain barrier, which we speculate may be related to the different pathogenesis of the two diseases. The pathogenesis of LBD is mainly the abnormal aggregation of alpha-synuclein in the brainstem and cortex, while AD mainly manifests as progressive memory loss, mainly due to the deposition and destruction of Aβ and tau proteins in the brain. The effect of LBD on cerebrospinal fluid circulation and the blood‒brain barrier was not as significant as that of AD. In addition, although CSFP is associated with CSFP pathological markers of AD, the impact on cognition in LBD patients may not be significant. Therefore, the changes in CSFP are not as significant as those in patients with AD.

## Limitations

First, all diagnoses were based on standardized clinical evaluation rather than pathological confirmation. Second, the study sample was relatively small; additionally, the study only evaluated the relationship between CSFP and cognition in patients with LBD and dementia patients with AD and did not include patients with other cognitive disorders. Third, the Qalb was used to evaluate the permeability of the BBB, but the destruction of the BBB is also affected by other substances, and neuroimaging (such as dynamic contrast-enhanced magnetic resonance imaging) is needed to accurately assess the extent of BBB disruption.

## Conclusion

In the dementia population with AD, the decrease in CSFP is related to more severe dementia and may be associated with further disruption of the BBB and depletion of the cerebrospinal fluid circulation, which influences the deposition of AD pathological markers and further affects the patient’s cognitive level. No such relationship was found in patients with LBD. A prospective study is needed to determine the relationship between the role of CSFP in the progression of cognitive disorders and patients with other types of cognitive disorders. It is suggested to conduct a randomized clinical trial to test whether the cognitive function of patients with AD can be improved by early manipulation of CSFP.

### Electronic supplementary material

Below is the link to the electronic supplementary material.

Supplementary Material 1

## Data Availability

The data that support the findings of this study are available from the corresponding author upon reasonable request.

## Abbreviations

MCI: Mild cognitive impairment
D-W test: Durbin-Watson test
CSFP: Cerebrospinal fluid pressure
AD: Alzheimer’s disease
LBD: Lewy body dementia
DLB: Dementia with Lewy bodies
PDD: Parkinson’s disease dementia
PD-MCI: Parkinson’s disease with mild cognitive impairment
MCI-LB: Mild cognitive impairment with Lewy bodies
MDRS: Mattis Dementia Rating Scale
MMSE: Mini-Mental State Examination
MoCA: Montreal Cognitive Assessment
SBP: Systolic blood pressure
CDR: Clinical Dementia Rating
BBB: Blood‒brain barrier
ICP: Intracranial pressure
IOP: Intraocular pressure
CI: Confidential interval
OR: Odds ratio
CT: Computed tomography
SD: Standard deviation
Qalb: Cerebrospinal fluid albumin to serum albumin ratio
RBD: Rapid eye movement sleep behavior disorder
EDS: Excessive daytime sleepiness
OSAHA: Obstructive sleep apnea hypopnea syndrome
ESS: Epworth Sleepiness Scale
PSQI: Pittsburgh Sleep Quality Index
HAMD: Hamilton Depression Inventory
HAMA: Hamilton Anxiety Inventory
